# Peer Case Management Promoting Advancement Along the HIV Care Continuum Among Black Men Who Have Sex with Men Living with HIV: Building Brothers Up

**DOI:** 10.1089/apc.2022.0105

**Published:** 2022-09-30

**Authors:** Kimberly A. Kisler, Jesse B. Fletcher, Cathy J. Reback

**Affiliations:** ^1^ Friends Research Institute, Inc., Los Angeles, California, USA.; ^2^ Department of Public Health, Rongxiang Xu College of Health & Human Services, California State University, Los Angeles, Los Angeles, California, USA.; ^3^ Department of Family Medicine, David Geffen School of Medicine, University of California, Los Angeles, Los Angeles, California, USA.; ^4^ Center for HIV Identification, Prevention and Treatment Services, Department of Family Medicine, University of California, Los Angeles, Los Angeles, California, USA.

**Keywords:** Black men who have sex with men (BMSM), HIV, HIV primary care, viral load suppression, peer case management

## Abstract

Black men who have sex with men (BMSM) in the United States are at elevated risk for HIV relative to their heterosexual and/or non-BMSM counterparts, yet on average demonstrate suboptimal HIV care linkage and rates of HIV primary care retention. From October 2019 to December 2020, 69 adult (i.e., aged 18–65) BMSM enrolled in *Building Brothers Up* (*2BU*), a 6-session peer case management intervention delivered across 3 months and designed to improve retention in HIV primary care through to full viral suppression. Peer case management sessions included detailed assessment of participants' needs and barriers to treatment, which led to the development of a participant-centered treatment plan. All participants self-identified as Black, about three-quarters self-identified as gay (72.5%), and 46.4% reported an annual income of $5000 or less. A total of 69 participants enrolled in *2BU*; however, multiply imputed chained equation logistic regressions were carried out on the final analytical data set (*n* = 40; 99 imputations) due to a large amount of COVID-19-related missing data. Although analyses of retention and achievement of viral suppression did not reach full significance, the probability of a Type-II hypothesis testing error was high, and viral load results (adjusted odds ratio = 1.56; 95% confidence interval = 0.94–2.60; *p* = 0.08) suggested that increased attendance to peer case management sessions may be associated with improved odds of achieving full viral suppression among BMSM. The significant impact of national race-related civil unrest and the COVID-19 pandemic on the target population during implementation of *2BU* is underscored.

## Introduction

The CDC^1,2^ reports that relative to men who have sex with men (MSM) from other racial/ethnic backgrounds, Black men who have sex with men (BMSM) in the United States evidence higher HIV prevalence, incidence, lower rates of linkage to and retention in HIV care, and are less likely to have achieved full viral suppression. In addition, BMSM are less likely than other MSM to initiate combination antiretroviral therapy (ART) and adhere to a treatment regimen.^1–3^ The CDC further estimates that 20% of BMSM living with HIV are not yet aware that they are infected, a higher rate than is observed among non-BMSM. One in two BMSM are likely to contract HIV in their lifetime.^4^

As demonstrated in the Los Angeles County (LAC) Comprehensive HIV Plan 2017–2021,^5^ disparities between BMSM and MSM from other racial/ethnic backgrounds are accentuated in LAC. Evidence demonstrates that BMSM are the most HIV-impacted group in LAC, with an incidence rate of 18 per 1000 for adult BMSM (the highest rate of any comparable age group in LAC). In LAC, BMSM also fare worse than other MSM for HIV Care Continuum outcomes—only 65% of BMSM living with HIV are linked to HIV primary care (compared with an overall rate of 74% among MSM in LAC), 52% are retained in care (vs. 59% among MSM overall), and only 48% have achieved full viral suppression (vs. 61% among MSM overall).^5^ These data have led LAC to identify BMSM as a priority population in the Plan for Ending the HIV Epidemic in LAC.^6^

Rates of increased HIV prevalence among BMSM are fueled by several well-known and pronounced disparities, particularly in relation to social determinants of health.^7,8^ For example, a meta-analysis found that BMSM had twofold greater odds of being unemployed, of earning a low income, of having previous episodes of incarceration, and of having relatively lower educational attainment compared with other MSM in the United States,^3^ and each of these represents a structural barrier associated with increased HIV risk.^9,10^ These structural factors are rooted in the sociopolitical and economic systems within the United States, and various forms of racism including institutional and structural racism, intersectional stigma, and the maltreatment of minoritized persons within the health care systems have been cited as key barriers to the success of Ending the HIV Epidemic within racial/ethnic minority populations broadly, and the scale-up of HIV prevention efforts such as pre-exposure prophylaxis interventions, specifically.^11^

Evidence has further demonstrated that increased rates of undiagnosed HIV infection and deficits in HIV primary care observed among BMSM in the United States are the function of several interrelated and reinforcing behavioral health issues, including but not limited to substance use (especially before/during sex),^9,12–14^ undiagnosed and/or untreated mental health disorder(s),^15,16^ especially depression,^17,18^ poverty and food insecurity,^19^ exposure to violence/trauma,^20,21^ and prejudice/discrimination,^22–24^ each of which disproportionately affects communities of BMSM in the United States. Again, structural racism, specifically racial segregation across the United States, rather than individual-level characteristics (e.g., HIV risk factors), has been cited as a main driver behind disparities within communities of color for infectious disease transmission (e.g., HIV, COVID-19).^25^

Biomedical and behavioral interventions aimed at advancing BMSM through the HIV Care Continuum are unlikely to succeed if basic needs are not first met. Therefore, an approach that includes case management aimed at addressing the immediate structural challenges and behavioral health needs of BMSM can help resolve issues that contribute to lack of engagement and retention in HIV care, and reduced initiation of and adherence to ART.

*Building Brothers Up* (*2BU*) was an adaptation of the evidence-informed intervention *Youth-Focused Case Management (YCM) Intervention to Engage and Retain Young Gay Men of Color in HIV Care*.^26^ The original *YCM* intervention targeted young Black and Latino MSM between the ages of 13–25, and consisted of 24 months of psychosocial case management that focused on treatment education/adherence support and HIV risk reduction counseling at clinical sites. In addition, the original intervention was structured so that participants attended weekly case management sessions for the first 2 months and monthly sessions for the remaining 22 months for a full 2-year intervention, with case management being delivered by two bachelors-level case managers.

The *2BU* intervention was adapted to target adult BMSM between the ages of 18–65 years, an older and demographically different group than the original *YCM* intervention. Also, the authors of the *YCM* intervention suggested in the primary outcomes article that the dosage of case management sessions and the length of the intervention could both be reduced to more effectively serve such highly impacted populations.^26^ Thus, *2BU* was adapted to include fewer sessions that were delivered over a shorter period of time. Lastly, *YCM* utilized bachelors-level case managers, whereas *2BU* was adapted to use a peer case manager (PCM). Utilizing peers to facilitate engagement with and navigation of complex care systems has been shown to be a promising intervention for HIV care since the early 2000s.^27,28^

Intervention staff who practice cultural humility and who are able to navigate access to HIV testing, treatment, and other support services, especially those with similarities in life experiences, have been cited as especially important among BMSM.^29^ Further, the use of peers (e.g., peer support workers) in the provision of mental health services has shown promising results.^30,31^

The adapted *2BU* intervention was guided by a single main research question: Would the adapted *2BU* peer case management intervention be able to retain BMSM living with HIV in care so they could reach and sustain viral suppression as was evidenced in the original *YCM* intervention among a different population?

## Methods

### Participants

From October 2019 through December 2020, 69 BMSM were screened, provided informed consent, and were enrolled in *2BU*. The inclusion criteria for participation were as follows:

(1) Self-identified as a Black man who has sex with other men.

(2) Between the ages of 18 and 65 years.

(3) Confirmed HIV-positive serostatus.

(4) Not engaged in care (defined as: had not had two or more HIV medical care appointments at least 90 days apart in the past 12 months, and/or had not had an undetectable viral load in the past 12 months) or engaged in care but at risk for falling out of care [defined as: had been incarcerated within the past 12 months, and/or had been unemployed for at least 3 months within the last 12 months, and/or had experienced housing instability (defined as: in the past 12 months had spent at least one night in a homeless shelter or transitional shelter, and/or the street or other outdoor public place, and/or in an abandoned building, and/or in a car or other vehicle, and/or at a friend's or family member's on a temporary basis, and/or in a sober living or recovery program or drug treatment program, and/or in a jail or prison), and/or had been diagnosed with an STI in the past 12 months, and/or had little interest in doing things and/or had felt down, depressed, or hopeless within the past 2 weeks, and/or had five or more drinks in a day at least once within the past 12 months, and/or had used marijuana, another street drug, and/or prescription medication “recreationally” at least once in the past 12 months, and/or had a negative experience with an HIV health care provider or clinic staff in the past 12 months].

(5) Resided in LAC.

(6) Willing and able to provide informed consent.

(7) Willing and able to comply with project requirements. Other than confirmation of HIV-positive serostatus, all other criteria were self-reported. Three participants self-withdrew after enrollment due to no longer wishing to continue participation, and one participant was withdrawn by the principal investigator after enrollment due to threats and safety concerns. The final sample size was *N* = 69.

### Procedures

To ensure that enrollment targets were met and a diversity of participants were enrolled, five proven recruitment strategies were utilized including the following: (1) Online recruitment: online banner advertisements and digital flyers were placed through geo-mapping on websites and social media that target BMSM, and through local digital spaces (e.g., Lynx, Facebook, Instagram). (2) The project logo and flyer were placed in print media for BMSM or that BMSM read (e.g., a program for a local popular lesbian, gay, bisexual, transgender (LGBT) chorus, local ‘zine targeting BMSM). (3) Street- and venue-based outreach: *2BU* staff conducted street- and venue-based outreach where BMSM congregate such as bars, clubs, food lines, and public libraries. Outreach was utilized to build and maintain ongoing trust and rapport with the population and thereby recruit into the project. (4) Poster/flyer advertisement: project posters/flyers were posted at collaborating community-based organizations and other venues that contained details about how to contact the PCM for further information regarding the project. (5) Participant-incentivized snowball recruitment: current participants were incentivized to recruit a maximum of three potential new participants from their social, sexual, and/or drug-using networks. Of the aforementioned recruitment strategies, participant-incentivized snowball recruitment was the most successful resulting in 26 inquiries. In addition, potential participants reported “word of mouth” by a friend as the most successful recruitment strategy resulting in 30 inquiries.

Following screening for eligibility and the informed consent process, potential participants were administered (either self-administered or by an intervention staff on a laptop or tablet due to pivots in evaluation protocols as a result of the COVID-19 pandemic) a baseline assessment that took ∼45 min to complete. Similar assessments were also given at 6 and 12 months post-enrollment. Participants were compensated with a $50 gift card at the completion of the baseline, 6-month, and 12-month follow-up assessments, and a $20 gift card at the completion of each local brief assessment (an additional assessment provided at each peer case management session). In addition, participants who referred a potential participant to *2BU* received a small gift (e.g., hygiene kit, snacks; valued at ∼$2) when the potential participant screened, and a $20 gift card if the potential participant was eligible and enrolled, for a maximum of three eligible and enrolled participants per active participant.

The total amount a participant could earn for enrolling and participating in the study was $330.

Enrollment closed on December 31, 2020, while follow-up assessments continued through December 31, 2021, allowing for the newest enrolled participants to be eligible for both the 6- and 12-month assessments. The project was conducted at Friends Community Center in Hollywood, CA, the community research site of Friends Research Institute, Inc. All study procedures were approved by the Western Institutional Review Board.

### The *2BU* intervention

**FIG. 1.**
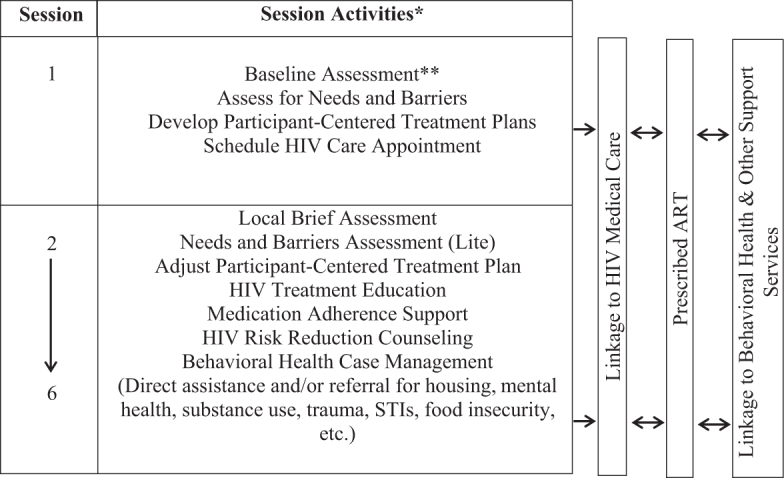
Overview of intervention delivery system. *Sessions occurred weekly in the first month (sessions 1–4), and monthly in the second and third months (sessions 5–6). **Baseline assessment included Patient Survey, Local Evaluation, and Local Brief Evaluation.

*2BU* was adapted to be delivered across 3 months and included a total of six sessions (sessions 1–4 were delivered weekly in month 1, session 5 in month 2, and session 6 in month 3). See Fig. 1 for an overview of the intervention delivery system.

The initial session included a detailed assessment of the participant's needs and barriers using the Needs and Barriers Assessment (NBA), which was designed to be participant-centered, meaning the issues reviewed may or may not have been directly connected to HIV care. In addition, the NBA was tailored to be responsive to the unique cultural needs of BMSM (i.e., experiences with racism, concerns regarding faith and spirituality). Once key issues were identified, the PCM worked with the participant to develop a participant-centered treatment plan, which included both short-term and long-term goals that needed to be addressed by the next scheduled meeting. Both the participant and PCM identified goals and action steps required to help the participant meet identified needs and/or overcome identified barriers. For example, a participant may have agreed to attend a scheduled appointment, while the PCM may have agreed to research additional support services on behalf of the participant.

Once the participant-centered treatment plan was created, both the participant and PCM signed the agreement to show commitment to the plan. A priority of the first session was to schedule an HIV care appointment for the participant if the participant was not already linked and engaged in HIV care.

During sessions 2–6, the participant's needs and barriers were reviewed and reassessed using a reduced version of the NBA called the NBA-Lite, and both parties once again agreed to short-term action steps for the subsequent session. In addition, the long-term goals identified in the participant-centered treatment plan were revised, as needed. An integral component of all six sessions was the assessment of behavioral health and other support service needs and the delivery of directly linked referrals through a “warm hand-off” (i.e., the PCM directly linked the participant to a needed service through a phone call, an email, or an in-person appointment rather than only providing the participant with the name of an agency, clinic, or provider) to partnering agencies.

Since *2BU* was implemented at a community research site, rather than a clinical site, all HIV care and most behavioral health and other support services needed to be accessed at a partnering organization; therefore, the PCM spent considerable time assisting the participant with making appointments, arranging for transportation, ensuring all documentation and required forms were in place, and facilitating a warm hand-off to a staff member at the partnering agency.

### COVID-19 adaptations

Implementation of *2BU* began in October 2019 and continued uninterrupted with no further adaptations until the COVID-19 pandemic outbreak in March 2020. The original adapted *2BU* intervention and all evaluation procedures were delivered completely in-person at the community research site. However, once “safer-at-home” directives required all staff to transition to remote work and the brick-and-mortar site closed during the lockdown, *2BU* and the associated evaluation procedures were pivoted to a fully remote/online (i.e., over the phone, on Zoom) modality of delivery. When the community research site intermittently opened in July 2020, *2BU* again pivoted to a hybrid design that allowed participants to participate in intervention and evaluation activities in-person, remotely/online, and/or a combination of the two delivery modalities.

### Measures

#### Patient survey

The digital Patient Survey (PS), developed by the Evaluation and Technical Assistance Provider (ETAP) at NORC at the University of Chicago, gathered information in the following six domains: (1) health attitudes and behaviors, (2) HIV care, (3) HIV medications and viral load, (4) behavioral health care/supportive services [specifically, the Personal Health Questionnaire Depression Scale (PHQ-8)], (5) experiences with health services, and (6) demographics. The ETAP developed the survey using validated questions and response items tailored to be culturally appropriate for BMSM. To minimize burden on clients and avoid respondent fatigue, the PS was designed to take ∼25 min to complete.

### Analysis

Outcome variables were (1) retention in HIV primary care (defined as: at least two HIV-related medical visits at least 90 days apart in the past 12 months, with at least one visit being with a provider with prescribing privileges), and (2) achievement of full viral suppression (defined as: a viral load of <200 copies/mL during their most recent viral load test in the past 12 months), both derived from the participant's electronic health records and/or clinic records. Statistical comparisons of observed rates of retention in HIV primary care and achievement of viral suppression over time were carried out using bivariate z-tests for differences in proportions.

Logistic regressions were carried out on the multiply imputed data sets (*n* = 99 imputations) using the PROC LOGISTIC and PROC MIANALYZE commands in SAS 9.4. The exogenous predictor was attendance to the peer case management sessions (range = 1–6). Participant age, baseline retention/viral load suppression (one each for the retention/viral suppression analyses, respectively), and baseline PHQ-8 scores were included as statistical controls in the final analytical models. We accounted for missing data using multiple imputation with chained equations and estimated the model and results by imputation to adjust for the uncertainty of missing data on the estimated standard errors of our estimates.

## Results

**FIG. 2.**
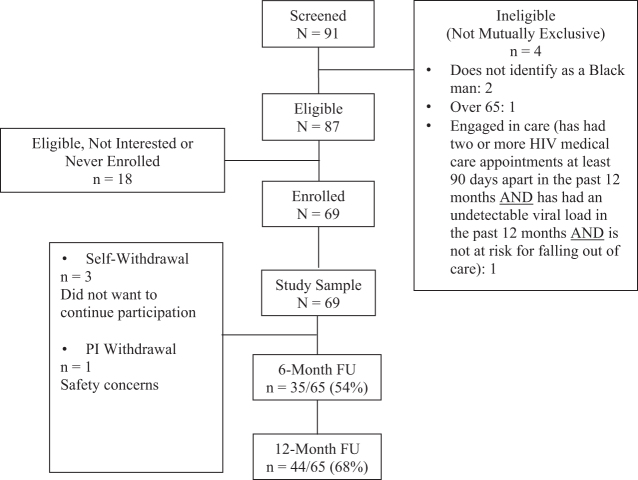
CONSORT diagram of study progression and retention.

Figure 2 illustrates the progress and retention from initial screening through 12-month follow-up assessments. A total of 91 potential participants were screened, 87 screened eligible, and 69 participants enrolled in *2BU*. Total participant follow-up rates were 54% at 6 months and 68% at 12 months.

Table 1 provides participants' sociodemographic characteristics. Participants were an average of 44.7 years of age (range = 18–65), all self-identified as Black and a minority, one-fifth also identified as Latinx (13.8%). Nearly three-quarters (72.5%) self-identified as gay. At baseline, nearly half (46.4%) reported an annual income of $5000 or less, the majority had at least a high school education or GED (36.8% high school graduate/GED, 38.2% more than a high school degree), and nearly three-quarters (72.5%) reported having Medicaid as their primary health insurance.

**Table 1.** Participant Sociodemographics and Intervention Attendance

	Mean or *N*	(SD) or %
Age (range = 18 − 65; median: 45)^a^	44.7	(11.7)
Ethnicity^b^		
Latinx-identified	8	13.8%
Not Latinx-identified	50	86.2%
Sexual identity^c^		
Gay-identified	50	72.5%
Not gay-identified	19	27.5%
Annual income^c^		
$5000 or less	32	46.4%
$5001–$10,000	12	17.4%
$10,001–$20,000	15	21.7%
More than $20,000	10	14.5%
Education^d^		
Less than high school diploma/GED	17	25.0%
High school graduate/GED	25	36.8%
More than high school graduate	26	38.2%
Patient Health Questionnaire Score^a^		
PHQ-8 score	13.5	(6.2)
Health insurance^c^ (categories not mutually exclusive)		
Covered CA/CA Healthcare Exchange	10	14.5%
Medicare	10	14.5%
Medicaid/Medi-Cal	50	72.5%
Ryan White	9	13.0%
Other insurance	9	13.0%
None	1	1.4%
Attendance to *2BU* sessions^c^ (range = 1 − 6; median = 5)	4.4	(1.6)

^a^ *N* = 70.

^b^ *n* = 58.

^c^ *n* = 69.

^d^ *n* = 68.

*2BU*, *Building Brothers Up*. PHQ-8, Personal Health Questionnaire Depression Scale; SD, standard deviation.

Table 2 provides participants' observed HIV Care Continuum outcomes at each study time point. Participants both significantly increased retention in HIV primary care and achievement of viral suppression at the 6-month time point, but no other longitudinal improvements were observed.

**Table 2.** Participant Retention in HIV Primary Care and Achievement of Viral Suppression by Time Point

	Baseline (*N* = 70)	6-Month follow-up (*n* = 47)	12-Month follow-up (*n* = 40)
	Mean (SD) or* N *(%)	Mean (SD) or* N *(%)	Mean (SD) or* N *(%)
Retention in HIV primary care	—	35 (74.5%)	34 (85.0%)
Achievement of viral suppression	26 (37.1%)	28 (59.6%)	20 (50.0%)

SD, standard deviation.

Table 3 regresses these same HIV Care Continuum outcomes (i.e., retention in HIV primary care, achievement of viral suppression in the past 12 months) onto participants' level of intervention attendance and study time point while controlling for baseline retention/viral suppression (respectively), participant age, and PHQ-8 score. Increased attendance to the *2BU* peer case management sessions was not associated with significantly increased odds of retention in HIV primary care [adjusted odds ratio (aOR) = 2.31; 95% confidence interval (CI) = 0.82–6.51; *p* = 0.11], although results were trending in the expected direction. The association between attendance to the peer case management sessions and odds of achieving full viral suppression was trending toward significance (aOR = 1.56; CI = 0.94–2.60; *p* = 0.08).

**Table 3.** Logistic Regressions of Retention in HIV Care and Achievement of Viral Suppression on Attendance to *Building Brothers Up* Sessions (Data Multiply Imputed, 99 Imputations; *n* = 40)

	Retention in HIV care	Viral suppression
	aOR (95% CI)	aOR (95% CI)
Attendance to *2BU* sessions	2.31 (0.82 − 6.51)	1.56 (0.94 − 2.60)^*^

Statistical controls: participant age, baseline retention/viral load suppression (respectively), PHQ-8 score.

^*^ *p* = 0.08.

*2BU*, *Building Brothers Up*; aOR, adjusted odds ratio; CI, confidence interval; PHQ-8, Personal Health Questionnaire Depression Scale.

## Discussion

Despite a national goal to End the HIV Epidemic by 2030,^32^ progress among BMSM has lagged. Disparities in social determinants of health that create structural barriers associated with increased HIV risk^3,7–9^ coupled with behavioral health issues, such as depression, fuel the epidemic among BMSM.^10,14,15,17,18,20–22,33^ Specifically, BMSM evidence lower rates of retention in HIV care and achievement of full viral suppression when compared with other MSM.^1,2^

Results demonstrated that using a case management approach may have allowed the participants to overcome acute barriers to ART adherence so that achievement of full viral suppression was possible. Indeed, many barriers identified in the participant-centered treatment plan and addressed across the six sessions were not directly related to HIV treatment and care, rather the sessions focused on issues such as housing instability, food insecurity, and employment options. Addressing these immediate and pressing challenges may have then enhanced the participants' abilities to shift their attention to addressing HIV-related needs.

Staffing *2BU* with a PCM, rather than bachelors-level case managers, may have also helped participants advance along the HIV Care Continuum. Community health workers who liaise between participants/patients and medical, behavioral health, and support service professionals have been found to play a pivotal and unique role in helping people living with HIV to sustain in HIV care.^34^ Further, BMSM have reported that receiving information on HIV and engaging in HIV care is more desirable if intervention staff are at a minimum practicing cultural humility, and at best have shared life experiences.^29^ The value of utilizing peer staff may have been accentuated during this time in history when the national conversation on race was centered on Black men as a result of the murder of George Floyd and other Black Americans.

In addition, *2BU* emphasized accessing behavioral health care as a potential goal within the peer case management sessions, and attention to mental health may have been especially important during the implementation period due to the COVID-19 pandemic and national response to the murder of George Floyd and others. Psychological well-being and substance use have been associated with increased HIV risk behaviors among BMSM,^35^ and depression and anxiety have been found to be more common among BMSM, particularly young BMSM^15^ than young White MSM, and people living with HIV than other adult populations.^36^ Substance use has also been demonstrated at high rates among BMSM.^37^

These behavioral health issues have been shown to prevent advancement along the HIV Prevention Continuum for BMSM^15^ as well as adherence to both HIV care and medication for people living with HIV generally,^36,38–40^ and BMSM specifically.^2^ Participants may have been more likely to access behavioral health services under the case management model and during a time of heightened need for mental health support, thus reducing barriers to HIV care and treatment. Further, the use of a peer staff may have once again helped to reduce the stigma associated with behavioral health, which allowed for open conversations, support, assessment of urgent needs, and timely referrals to behavioral health services. Peer staff who have experienced their own mental health issues have been shown to make unique contributions to patients' recovery, including promoting hope, enhancing a belief in recovery, and increasing self-esteem, self-efficacy, and self-management of challenges.^30,31^

While these results are promising, outcomes must be considered within the project's limitations. Given that *2BU* was not a randomized controlled trial with a standard-of-care comparison group, results cannot be exclusively contributed to the *2BU* intervention. Also, this study utilized a convenience sample of BMSM living with HIV who self-selected to participate in this peer case management intervention. As a result, findings presented here may not be generalizable to other samples of BMSM. Further, the amount of missing data present in this study, even after the application of multiple imputation, must inevitably raise concerns about the generalizability of the findings discussed, as well as the significant likelihood of Type-II hypothesis testing errors.

Most importantly, *2BU* began implementation before the COVID-19 pandemic and much of the implementation period occurred during the initial wave of the pandemic. Abrupt closures of the community research site where the intervention was delivered and the resulting pivots to remote/online intervention and evaluation delivery modalities, which may have reduced the accessibility to the intervention and evaluation procedures (i.e., reliable Wi-Fi, lack of familiarity with how to use Zoom or email), coupled with the significant challenges associated with the pandemic, impacted the experiences of the participants.

In addition, many health care, behavioral health, and other support service providers paused services as a result of the pandemic, which means accessing services through the peer case management intervention may have been more difficult (or even impossible) during a large portion of the implementation period. Retention in HIV care may have been especially challenging if participants were unable to attend in-person appointments and/or if they felt less comfortable with or even unable to access telehealth appointments, which would have directly impacted the *2BU* intervention.

Despite these significant limitations, results presented here provide preliminary evidence that peer case management interventions may assist BMSM, a population significantly impacted by HIV, in reaching full viral suppression.
